# Aggregated *Mycobacterium tuberculosis* Enhances the Inflammatory Response

**DOI:** 10.3389/fmicb.2021.757134

**Published:** 2021-12-02

**Authors:** Hylton E. Rodel, Isabella A. T. M. Ferreira, Carly G. K. Ziegler, Yashica Ganga, Mallory Bernstein, Shi-Hsia Hwa, Kievershen Nargan, Gila Lustig, Gilla Kaplan, Mahdad Noursadeghi, Alex K. Shalek, Adrie J. C. Steyn, Alex Sigal

**Affiliations:** ^1^Africa Health Research Institute, Durban, South Africa; ^2^Division of Infection and Immunity, University College London, London, United Kingdom; ^3^Ragon Institute of MGH, Harvard, and MIT, Cambridge, MA, United States; ^4^Department of Chemistry, Institute for Medical Engineering and Sciences, MIT, Cambridge, MA, United States; ^5^Broad Institute of MIT and Harvard, Cambridge, MA, United States; ^6^Koch Institute for Integrative Cancer Research, MIT, Cambridge, MA, United States; ^7^University of Cape Town, Cape Town, South Africa; ^8^School of Laboratory Medicine and Medical Sciences, University of KwaZulu-Natal, Durban, South Africa; ^9^Department of Microbiology, Centres for AIDS Research and Free Radical Biology, University of Alabama at Birmingham, Birmingham, AL, United States; ^10^Max Planck Institute for Infection Biology, Berlin, Germany

**Keywords:** *Mycobacterium tuberculosis*, TB pathogenesis, aggregation, inflammation, TNF-alpha, phagocytosis

## Abstract

*Mycobacterium tuberculosis* (Mtb) bacilli readily aggregate. We previously reported that Mtb aggregates lead to phagocyte death and subsequent efficient replication in the dead infected cells. Here, we examined the transcriptional response of human monocyte derived macrophages to phagocytosis of aggregated Mtb relative to phagocytosis of non-aggregated single or multiple bacilli. Infection with aggregated Mtb led to an early upregulation of pro-inflammatory associated genes and enhanced TNFα signaling via the NFκB pathway. These pathways were significantly more upregulated relative to infection with single or multiple non-aggregated bacilli per cell. Phagocytosis of aggregates led to a decreased phagosome acidification on a per bacillus basis and increased phagocyte cell death, which was not observed when Mtb aggregates were heat killed prior to phagocytosis. Mtb aggregates, observed in a granuloma from a patient, were found surrounding a lesion cavity. These observations suggest that TB aggregation may be a mechanism for pathogenesis. They raise the possibility that aggregated Mtb, if spread from individual to individual, could facilitate increased inflammation, Mtb growth, and macrophage cell death, potentially leading to active disease, cell necrosis, and additional cycles of transmission.

## Introduction

*Mycobacterium tuberculosis* (Mtb) infection of a human lung results in either a latent disease state, in which an individual remains asymptomatic, or active tuberculosis (TB) disease, manifesting as hemoptysis, lung damage, weight loss, and other severe systemic effects (Russell, 2001, 2007; Barry et al., 2009; Ramakrishnan, 2012; Martin et al., 2017). In active TB, Mtb infection leads to necrosis of the granuloma, the structure which contains Mtb infection, attempting to isolate it from the surrounding lung (Kaplan et al., 2003; Barry et al., 2009; Lin et al., 2014; Lenaerts et al., 2015). This in turn results in the necrotic granuloma undergoing liquefaction that can breach the alveoli, entering the airway and is followed by expectoration of bacilli out of the lung and transmission to other hosts (Russell, 2001, 2007; Barry et al., 2009; Ramakrishnan, 2012).

The host–pathogen interactions that tip the balance to active disease are not clearly defined. Aggregation has been proposed to be associated with pathogenicity (Orme, 2014; Tezera et al., 2020) and increased Mtb virulence in mice and *ex vivo* (Glickman et al., 2000; Ufimtseva et al., 2018; Arias et al., 2020). Mtb aggregates have been observed in human lung tissue (Kaplan et al., 2003), though the fraction of bacilli in this state is unclear. Additionally, Mtb aggregates have recently been shown to be transmitted in bio-aerosols (Dinkele et al., 2021).

We have previously demonstrated using time-lapse microscopy that infection with aggregated Mtb preferentially leads to macrophage death (Mahamed et al., 2017) and this has been observed by others using different methods (Lee et al., 2006; Brambilla et al., 2016). Death of infected macrophages in turn results in rapid replication of the bacilli inside the dead infected cells (Mahamed et al., 2017), an observation which was independently confirmed (Lerner et al., 2017). The necrotic, infected cell may then be phagocytosed by another macrophage, leading to additional cycles of host cell death and bacterial growth that may close a positive-feedback loop (Mahamed et al., 2017), provided more phagocytes are recruited to the infection area.

Macrophages show extensive transcriptional remodeling of their immune and inflammatory pathways (Jenner and Young, 2005; Rothchild et al., 2019; Pisu et al., 2020). This includes upregulation and secretion of TNFα, IL8, CCL3, CCL4, IL1β and other factors involved in inflammation (Ragno et al., 2001; Volpe et al., 2006; Roy et al., 2018). This response activates macrophages for the killing of phagocytosed Mtb (Roca and Ramakrishnan, 2013) and recruits additional cell types, such as neutrophils, to the site of Mtb infection (Zhang et al., 1995; Nandi and Behar, 2011). While such a response may be host protective, macrophage cell death may also be a consequence (Tobin et al., 2012; Roca and Ramakrishnan, 2013; Tezera et al., 2020). Here, we investigated the effects of Mtb aggregation on the macrophage early transcriptional response. We observed that infection with Mtb aggregates led to a stronger early inflammatory response in human monocyte derived macrophages, with higher secretion of TNFα, as well as upregulation of genes leading to chemotaxis. We also observed that Mtb aggregates accounted for a substantial number of the Mtb identified on the periphery of a cavitary lesion. Taken together, these results may be consistent with Mtb aggregation playing a role in TB pathogenesis.

## Materials and Methods

###  Macrophage Cultures

Peripheral blood mononuclear cells were isolated by density gradient centrifugation using Histopaque 1077 (Sigma-Aldrich, St Louis, MO). CD14+ monocytes were purified under positive selection using anti-CD14 microbeads (Miltenyi Biotec, San Diego, CA, USA). For RNA-Seq protocols, CD14+ monocytes were seeded at 1 × 10^6^ cells per well in non-tissue culture treated 35 mm 6-well plates. For timelapse microscopy protocols, CD14+ monocytes were seeded at 0.2 × 10^6^ cells per 0.01% fibronectin (Sigma-Aldrich) coated 35 mm glass bottom optical dishes (Mattek, Ashland, MA, USA). Monocytes were then differentiated in macrophage growth medium containing 1% each of HEPES, sodium pyruvate, L-glutamine, and non-essential amino acids, 10% human AB serum (Sigma-Aldrich), and 50 ng/ml GM-CSF (Peprotech, Rocky Hill, NJ) in RPMI. The cell culture medium was replaced 1, 3, and 6 days postplating.

###  Mtb Culture and Macrophage Infection

The mCherry fluorescent strain of H37Rv Mtb was derived by transforming the parental strain with a plasmid with mCherry under the smyc' promoter (gift from D. Russell). Mtb were maintained in Difco Middlebrook 7H9 medium enriched with oleic acid-albumin-dextrose catalase supplement (BD, Sparks, MD). Three days before macrophage infection, Mtb were switched to grow in Tween 80-free media. On the day of infection, exponentially growing bacterial culture was pelleted at 2,000 × g for 10 min, washed twice with 10 ml PBS, and large aggregates broken up by shaking with sterilized 2–4 mm glass beads for 30 s (bead beating). Note that 10 ml of PBS was added and large clumps were further excluded by allowing them to settle for 5 min. Where heat killing was required, Mtb suspension was placed in a heating block for 20 min at 80°C. To generate single Mtb bacilli, the bacterial suspension was passed through a 5 μm syringe filter following aggregate preparation. The resulting singlet and aggregate Mtb suspensions were immediately used to infect differentiated monocyte-derived macrophages (MDMs). Mtb grown in media containing Tween 80 surfactant was grown in parallel with detergent free Mtb culture to monitor bacterial growth and calibrate macrophage infection using optical density readings. MDMs were infected with 150 μl Mtb aggregate suspension or 1,000 μl singlet suspension for 3 h, washed with PBS to remove extracellular Mtb, and incubated for a further 3h.

###  Cell Sorting

Following infection, macrophages were lifted from non-tissue culture treated plates, using 1 ml of Accutase (Sigma-Aldrich) cell dissociation reagent per 35 mm well, and transferred to FACS tubes. Note that 1 μl of Draq7 was added per macrophage containing FACS tube. Macrophage populations were gated into high and low Mtb infected populations based on Mtb mCherry fluorescence within each macrophage (measured at 561 nm). The number of bacilli per infected macrophage in each gate was estimated by comparing fluorescence distributions of Mtb in FACS data to the corresponding Mtb fluorescence data in microscopy (Supplementary Figure 1), as we had previously shown a tight correspondence between bacterial CFU and fluorescence (Mahamed et al., 2017, Figure 1; Supplementary Figure 2). Live and infected macrophages were selected for by gating out dead cells with compromised membranes using Draq7 fluorescence (at 633 nm excitation). Note that 10,000 infected macrophages, per tube, were sorted into Trizol (Thermo-Fischer) and snap frozen in a dry ice and 99% isopropanol slurry using a BD FACSAria III flow cytometer (BD, New Jersey, USA).

**Figure 1:**
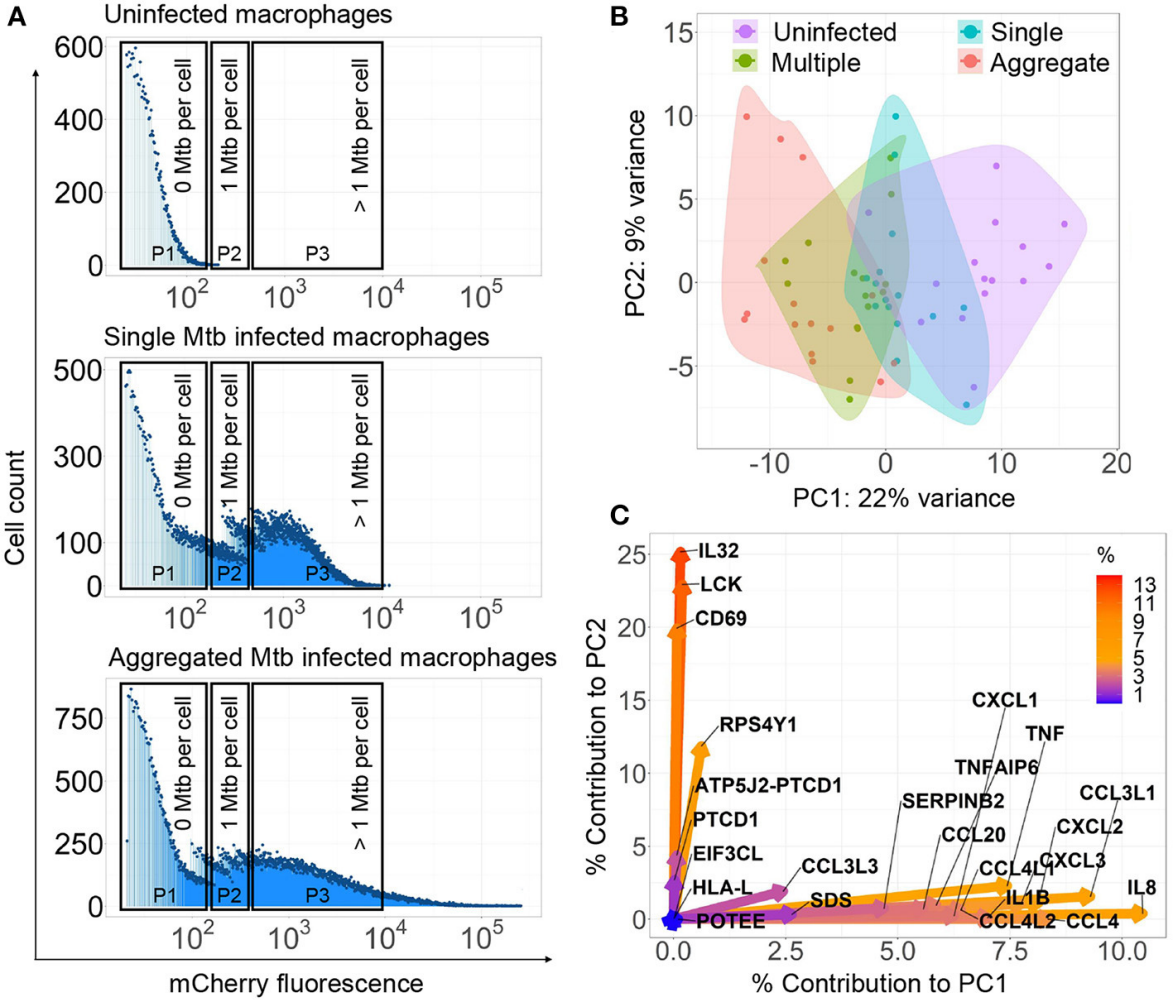
*Mycobacterium tuberculosis* (Mtb) aggregation changes the macrophage transcriptional response. **(A)** Monocyte-derived macrophages (MDMs) were infected with mCherry labeled, aggregated Mtb (bottom panel), single Mtb bacilli (middle panel), or uninfected (top panel) and sorted for RNA-Seq 3 h post-infection. Populations consisted of cells infected with aggregated Mtb (Gate P3—bottom panel), cells infected with single or few bacilli (Gate P2—middle panel), cells infected with multiple single bacilli (Gate P3—middle panel), or uninfected (Gate P1—top panel). Dead cells were excluded. X-axis on histograms is signal from mCherry fluorescent Mtb in MDMs, y-axis is count. **(B)** Principal component analysis (PCA) of the top 0.1% most variably expressed genes following rlog normalization and batch correction in R. Small circles are individual experiments (3 repeats from each of 5 blood donors). **(C)** Percentage contribution of individual genes used in the PCA. Color bar represents the percent contribution of individual genes to the first two principal components.

###  RNA-Seq

Snap frozen samples were stored at −80°C prior to transport and sequencing. Sample cDNA libraries were prepared according to protocols established by Trombetta et al. (2014) and sequenced on an Illumina NextSeq 500/550 instrument. Transcripts were aligned to human reference hg19 and read count libraries generated using the RSEM software package. Fastq files were uploaded to the NCBI GEO under the accession number GSE173560.

###  Transcriptomics Data Analysis

Read count libraries, generated for each of the 15 replicates per treatment at an average read depth of 4 per base (Lander and Waterman, 1988), were processed using the DESeq2 package for the R programming platform (Love et al., 2014). Metadata and read count matrices from each batch were concatenated into a single metadata and read count matrix prior to processing. For principal component analyses (PCA) analyses, count matrices were R-log normalized in DESeq2, to prevent low abundance transcripts from dominating variation, and corrected for batch effects using the SVA ComBat function for R. Read count matrices were then arranged in descending order by variance across treatment conditions, and the top 0.1% of variable genes plotted in a PCA to reveal any clustering evident at the early assay timepoint. For differential expression analysis, the conventional DESeq2 negative binomial model was used, including blood donor as a factor. Candidate genes identified in this way were cross-referenced with the top 1% of R-Log normalized, variance ordered lists to narrow the list of potential candidate genes. Individual genes were then tested for significant differences between infection conditions using a Hochberg corrected Kruskal–Wallis U-test.

###  Cytokine Analysis

MDMs were isolated and differentiated as previously described, at a concentration of 1 × 10^6^ cells per well on tissue culture treated 35 mm 6-well plates. MDMs were infected with 150 μl Mtb aggregate suspension or 1,000 μl Mtb single suspension and incubated for 3 h before being washed with PBS to remove extracellular Mtb, and incubated for a further 3 h. Supernatant was then collected, 0.2 μm filtered, and frozen prior to cytokine quantification. Cytokine levels were quantified using a Biorad Bioplex 200 (BioRad, California, USA) instrument and custom R&D systems Luminex cytokine panel kit, according to kit instructions. Custom panels were constructed to have a broad array of cytokines to validate against transcriptional data. Due to the early supernatant harvesting timepoint, only cytokines that had levels above background were retained for analysis purposes.

###  Microscopy

Macrophages and bacteria were imaged using an Andor (Andor, Belfast, UK) integrated Metamorph-controlled (Molecular Devices, Sunnyvale, CA, USA) Nikon TiE motorized microscope (Nikon Corporation, Tokyo, Japan) with a 20x, 0.75 NA phase objective. Images were captured using an 888 EMCCD camera (Andor). Temperature and CO_2_ were maintained at 37°C and 5% using an environmental chamber (OKO Labs, Naples, Italy). For timelapse protocols, images were captured once every 10 min for the duration of the time-lapse. For each acquisition, images were captured at wavelengths applicable to fluorophores used in the analysis including transmitted light (phase contrast), 561 nm (RFP), and 640 nm (DRAQ7, lysotracker). Image analysis was performed using custom written matlab script. Single cell segmentation was manually carried out prior to fluorescent signal quantification. For each cell, fluorescent signal in each channel was quantified as pixel intensity.

###  Macrophage Acidification Assay

Single cell fluorescence data for lysotracker acidification was acquired at a single timepoint at 6 h postinfection using the confocal microscopy system described previously. MDMs on fibronectin-coated optical dishes were infected with 400 μl Mtb aggregate suspension and incubated for 3 h before being washed with PBS to remove any cell-free Mtb and further incubated for 2 h. One hour before image acquisition, lysotracker (Thermofisher, Waltham, MA, USA) was added to wells at a concentration of 75 nM. Images were processed as previously described to acquire pixel fluorescence intensity data for each fluorescent channel per cell. Model fit was to 3/*r*, where *r* was aggregate radius.

###  Combination Staining of Human Lungs

Human lung tissue were cut into 2 mm thick sections and picked on charged slides. Slides were baked at 56°C for 15 min. Mounted sections were dewaxed in xylene followed by rinse in 100% ethanol and 1 change of SVR (95%). Slides were then washed under running water for 2 min followed by antigen retrieval via heat-induced epitope retrieval (HIER) in Tris-sodium chloride (pH 6.0) for 30 min. Slides were then cooled for 15 min and rinsed under running water for 2 min. Endogenous peroxide activity was blocked using 3% hydrogen peroxide for 10 min at room temperature (RT). Slides were then washed in PBST and blocked with protein block (Novolink) for 5 min at RT. Sections were incubated with primary antibodies for CD68 (M0814-CD68-KP1, DAKO, 1:3000) followed by washing and incubation with the polymer (Novolink) for 30 min at RT. Slides were then washed and stained with DAB for 5 min, and washed under running water for 5 min. For combination staining, slides were incubated with heated carbol fuchsin for 10 min and then washed in running tap water. Note that 3% acid alcohol was applied to the slide to decolorize for 30 s or until sections appeared clear. Slides were then washed in running tap water for 2 min and where then counterstained with methylene blue. Slides were rinsed under running water, dehydrated, and mounted in Distyrene Plasticiser Xylene (DPX).

###  Semi-automated Detection of Mtb in Histology Slides

RGB Images of resected lung tissue in .ndpi format were converted to .TIFF file types (without compression) to enable compatibility with Matlab (Mathworks, Massachusetts, USA) image processing functions. The resultant image files were sectioned into smaller tiles and imported individually into Matlab in a looped process. Saturated image tiles (tiles that had near completely white pixel values at all positions) were discarded from the analysis. Each layer of the RGB image matrix was converted to a grayscale image. Putative Mtb bacilli were then manually identified and used to set threshold values to eliminate background noise relative to Mtb signal. Thresholded images were then used to identify “Mtb-like” objects in an image for further downstream verification. Each of these objects was numerically labeled and compared against a user determined spectral profile to identify putative Mtb and eliminate false positives. Host cellular nuclei were identified in an identical fashion, but compared to a different spectral profile to identify true positives. Mtb and cellular nuclear objects identified in this way were tested for association, based on mean alveolar macrophage radius (Krombach et al., 1997), and added to a matrix that was mapped onto a reduced image of the whole tissue section to reveal physical locations of Mtb infection. Following automated Mtb object detection, each object was manually validated to ensure only putative detections were used for downstream analysis. Additionally, aggregated or single Mtb classification was manually validated. The resultant curated matrices were exported to R for graphing. Average single bacterium size was calculated by finding the mean area of manually validated Mtb single bacteria. Mean single Mtb area was then used to estimate the number of bacteria in all Mtb objects. The largest manually validated single Mtb object was used as a threshold above which all other Mtb objects were classified as aggregates.

## Results

###  Aggregated Mtb Shows a Distinct Transcriptional Response Dependent on Both Mtb Number and Aggregation State

We examined whether bacterial aggregation could differentially modulate the early host cell transcriptional response. We infected MDM with single or aggregated bacilli expressing the fluorescent mCherry protein (3 repeats for each of 5 donors, *n* = 15). Single Mtb were obtained by bead beating Mtb grown in the absence of detergent, followed by filtration (see Materials and Methods section). At 3 h postinfection, we sorted live MDM which internalized aggregated Mtb (Figure 1A), or live MDM which internalized single Mtb or multiple single Mtb. As a control, we sorted uninfected MDM. We determined the range of fluorescence per single Mtb (Supplementary Figure 1) and we used sorting gates, which corresponded to zero Mtb per macrophage (Figure 1A, sorting gate P1), one Mtb per macrophage (Figure 1A, sorting gate P2), or more than one Mtb per macrophage (Figure 1A, sorting gate P3). Infection with single or aggregated Mtb led to subsets of infected macrophages in the P3 (>1 Mtb/cell) gate. Sorted macrophages were therefore classified as multiply infected by single Mtb if single Mtb was used as the source of infection, or as having internalized an Mtb aggregate if aggregated Mtb was used as the source of infection.

We performed population RNA-Seq on the different sorted populations. We identified the most variably expressed genes, indicating the strongest up- or downregulation after normalization and batch correction. We used PCA to plot the top 0.1 % of variable genes across the different conditions. We observed that the infection conditions could be separated along the first principal component, where there was a graded response in gene expression from uninfected to singly infected to multiple infected to aggregate infected cells (Figure 1B), indicating that both the number of infecting bacilli and the aggregation state determines the transcriptional profile. Principal component 1 contained 22% of total PCA variability and the genes contributing to this separation included TNF, IL8, CCL4, IL1β, CXCL2, CXCL3 and other genes involved in inflammation and chemotaxis (Figure 1C). Principal component 2 encompassed 9% of total PCA variability. Genes contributing to separation along this axis included IL32, CD69, and LCK, whose functions include upregulation of TNF-alpha (IL32) (Kim et al., 2005), and lymphocyte activation (CD69 and LCK) (Lopez-Cabrera et al., 1993; Vogel and Fujita, 1995).

**Figure 2:**
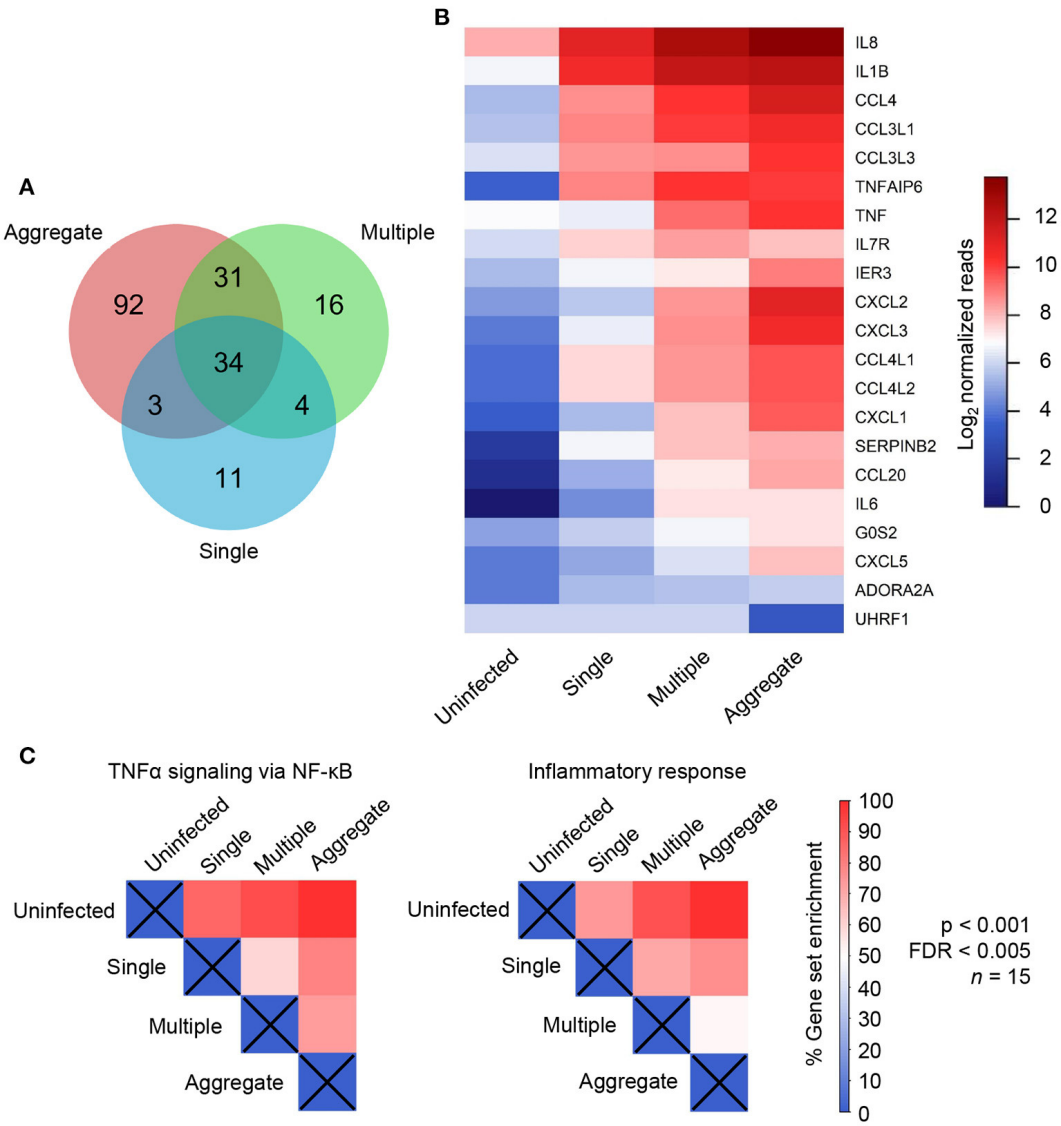
Individual genes and gene sets were differentially regulated in aggregate *Mycobacterium tuberculosis* (Mtb) infection. **(A)** Venn diagram showing the number of differentially regulated genes in single (blue), multiple (green) and aggregate Mtb (salmon) infected macrophages relative to uninfected macrophages as identified by DESeq2 differential expression analysis. **(B)** Heatmap showing read counts of genes identified as most variably expressed between infection conditions and selected by DESeq2 as significant (21 genes). **(C)** Normalized enrichment score (NES), expressed as percentage of maximum enrichment for the gene sets defined as “TNFα signaling via NF-κB” and “Inflammatory response.” Enrichment scores were calculated for all treatment comparisons and were significantly different at nominal *p* < 0.001 and FDR < 0.005, with the exception of the aggregate to multiple comparison for the “inflammatory response” gene set (where *p* < 0.05 and FDR = 0.24).

We quantified the number of genes differentially regulated between infection conditions using DESeq2 differential expression analysis. We compared each Mtb infection condition to the uninfected MDM control and generated lists of differentially regulated genes. These lists were used to generate a Venn diagram (Figure 2A). Aggregate-infected MDMs had the highest number of differentially regulated genes relative to uninfected MDMs at 160. Of these, 65 were shared with multiple Mtb-infected MDM, 37 with single-infected MDM, and 34 were common to all infection types. Multiple Mtb-infected MDMs had the next highest number of differentially regulated genes at 85 and single-infected MDMs the lowest number at 53. Note that 92 genes were uniquely regulated by aggregate infection. This was much higher than in single Mtb infection (11 unique) or infection with multiple single Mtb (16 unique).

We next examined the set of genes which were both selected as differentially regulated using DESeq2 (adjusted *p* < 0.1), and most strongly regulated, as seen by the highest variability in expression across conditions (top 1% of variable genes in PCA analysis). We then generated a read count heatmap across infection conditions using this refined set of genes. This resulted in a list of 21 genes including IL1β, IL8, IL6, CCL4, CXCL2, CXCL3, IER3, SERPINB2, and TNFAIP6 (Figure 2B, Supplementary Table 2) whose functions include TNFα response, inflammation, neutrophil chemotaxis, and regulation of apoptosis (Baggiolini and Clark-Lewis, 1992; Dickinson et al., 1995; Menten et al., 2002; Danchuk et al., 2011; Dinarello, 2018). We mostly observed upregulated expression, where upregulation increased from singly infected to multiply infected, to aggregate-infected MDM. There were two exceptions: IL7R, whose functions include cell survival and apoptosis (Leung et al., 2020). This gene was most strongly expressed in cells infected with multiple single Mtb. The other exception was UHRF1, which was strongly downregulated in aggregates but unaffected by the other infection types. UHRF1 is involved in cell cycle progression and depletion has been reported to induce cell-cycle arrest (Tien et al., 2011). These results indicate that aggregation results in a more pronounced cellular response across multiple genes relative to the other infection types, including infection by multiple single Mtb.

We next examined the TNFα and inflammatory responses over multiple genes. These responses had the highest enrichment score using gene set enrichment analysis (GSEA, see Supplementary Table 1). We observed that, relative to uninfected cells, there was enrichment of both gene sets when MDMs were infected with single Mtb. However, a further increase in the enrichment score occurred when infection was by multiple non-aggregated bacilli and was highest with aggregated bacilli. Interestingly, both the number of bacilli and aggregation state showed an effect: there was enrichment in both TNFα signaling and the inflammatory response with multiple non-aggregated Mtb relative to single Mtb, and in TNFα signaling with aggregate infection relative to infection with multiple non-aggregated bacilli per cell (Figure 2C). Taken together, there is a trend toward stronger expression of inflammatory mediators with a progression from single infection to infection with unaggregated multiple Mtb to aggregates.

**Figure 3:**
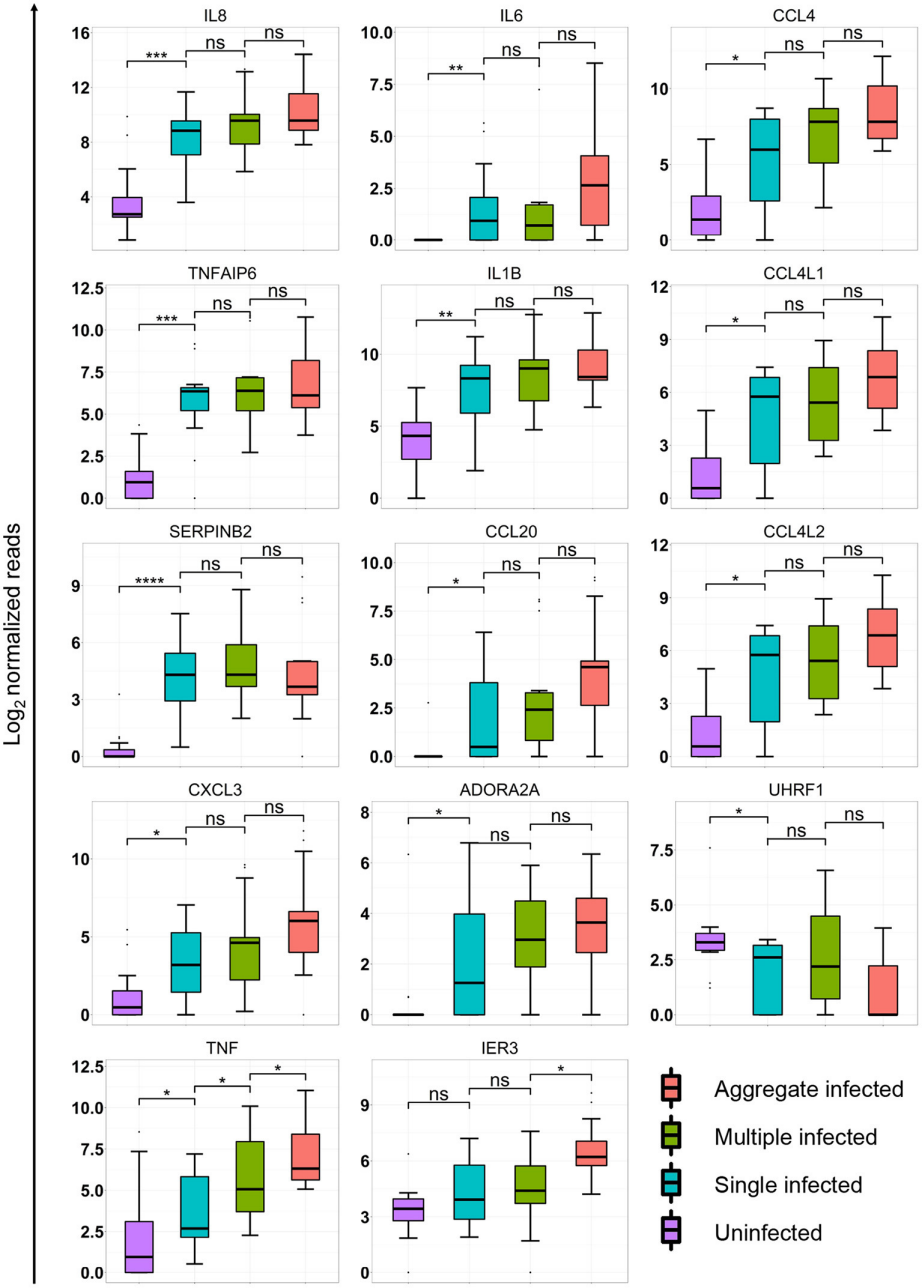
Aggregation state alters macrophage transcriptional response at the single gene level. Box plots of median and interquartile range of log normalized read counts from 15 independent infections of monocyte-derived macrophage (MDM) from 5 blood donors. Shown are expression levels as log transformed read counts in uninfected, single infected, multiple infected and aggregate infected macrophages. p-values are ns = not significant; * < 0.01; ** < 0.001; *** < 0.0001; **** < 0.00001; as determined by Kruskal–Wallace non-parametric test with Hochberg multiple comparison correction, with comparisons performed to nearest neighbor in graph (3 comparisons total).

We examined whether single genes identified in the refined list of candidates (Figure 2B) were significantly different between infection conditions. We performed multiple comparison corrected paired comparisons between uninfected and single, single and multiple, and multiple and aggregate infected conditions for each of the 21 identified genes. Of these genes, 14 showed significant differences in at least one of the comparisons (Figure 3). Two trends were identified. The first trend showed significant. transcriptional regulation regardless of number of infecting bacteria or aggregation state. TNFAIP6 and SERPINB2 followed this trend. The second trend showed transcriptional regulation that was most strongly influenced by the aggregation state. Most genes followed this trend with significance achieved in TNF and IER3 (Figure 3).

###  Aggregate Mediated Macrophage Death Requires Live Mtb

**Figure 4:**
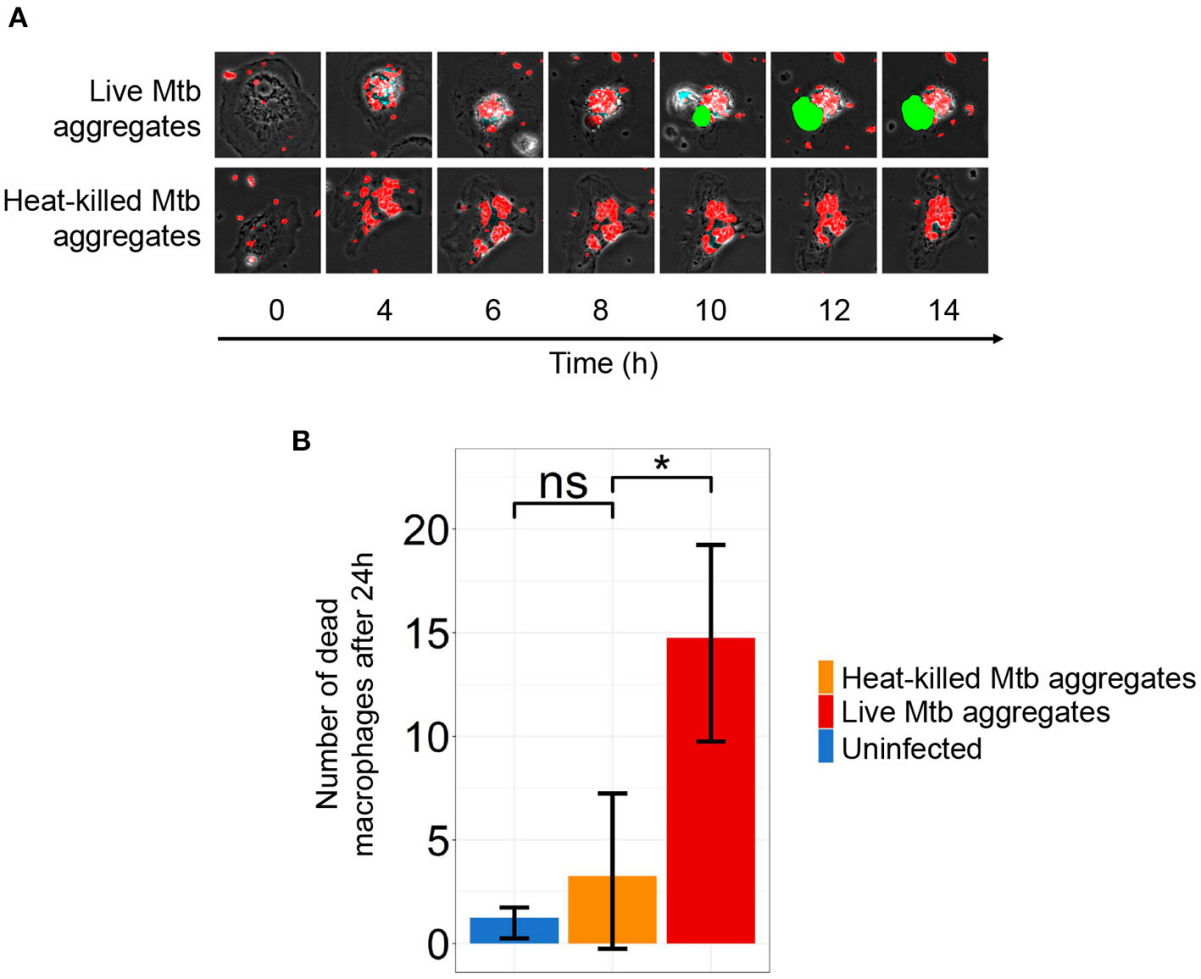
Macrophage death is dependent on infection with live *Mycobacterium tuberculosis* (Mtb) aggregates. **(A)** Timelapse microscopy showing mCherry labeled Mtb (red) induced monocyte-derived macrophage (MDM) death as detected by DRAQ7 (green). **(B)** The number of dead cells in MDM infected with live Mtb (red bar), heat-killed Mtb (orange bar), or uninfected (blue bar) after 24 h. Shown are mean ±SD of DRAQ7 positive cells after 24 h. *p*-values are ns = not significant and * < 0.01.

We tested whether macrophage cell death, elicited by Mtb aggregates, required the bacilli to be live. MDM death was quantified using confocal fluorescence timelapse microscopy to detect penetration of the cell membrane permeability dye DRAQ7, where penetration of dye is associated with the loss of plasma membrane integrity (Figure 4A).

We infected MDM with live Mtb aggregates (Figure 4A, Supplementary Movie 1) or with Mtb aggregates that had been heat-killed for 20 min at 80°C (Figure 4A, Supplementary Movie 2). We then monitored cell death in infected MDM over time. Despite the aggregates being heat killed, they were readily phagocytosed by macrophages (Supplementary Movie 2). We observed extensive MDM death when MDMs were infected with live Mtb aggregates. In contrast, the number of dead MDM infected with fluorescent, heat killed aggregates did not markedly differ from uninfected MDMs (Figure 4B).

###  Reduced Acidification of Bacilli in Intracellular Mtb Aggregates

**Figure 5:**
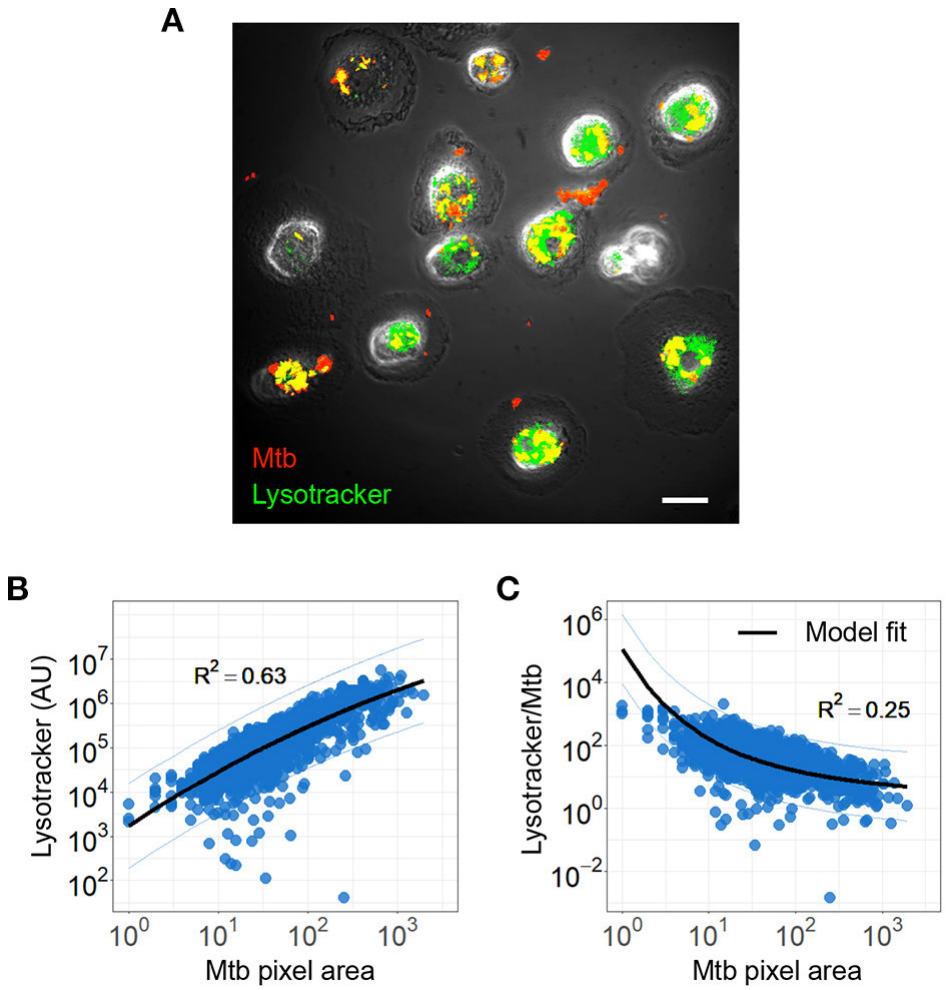
*Mycobacterium tuberculosis* (Mtb) acidification per Mtb bacillus decreases with increasing Mtb aggregate size. **(A)** Image of lysotracker (green) colocalization with phagocytosed mCherry expressing Mtb (red). Scale bar is 20 μm **(B)** lysotracker fluorescence as a function of total aggregate area. The linear regression line is shown in black (*R*^2^ = 0.63, *p* < 0.0001). **(C)** Ratio of lysotracker fluorescence to Mtb fluorescence as a function of Mtb area. Black line shows a model based on the surface area to volume ratio (*R*^2^ = 0.25, *p* < 0.0001, black line).

To examine the possible causes of sub-optimal control of intracellular Mtb aggregates, which may lead to macrophage death, we asked whether the aggregation state modulates the ability of the macrophages to acidify phagosome associated Mtb. We infected MDM with single or aggregated Mtb for 6 h, and imaged cells following staining with the LysoTracker reporter for acidification. We quantified Mtb and lysotracker fluorescence within each cell and the areas of overlap between Mtb and lysotracker fluorescence (Figure 5A).

We plotted lysotracker fluorescence against Mtb area and found that lysotracker signal increases with Mtb signal (Figure 5B). This shows that as the number of Mtb per macrophages increase, acidification per macrophage increases. However, when this signal is divided by the total number of bacilli internalized by the macrophage, the acidification per bacillus is lower as aggregate size increases (Figure 5C). This can be described by a model which accounts for the expected relationship of surface area to volume of a phagosome (see Materials and Methods section).

###  Mtb Aggregates at the Periphery of a TB cavity in the Human Lung

**Figure 6:**
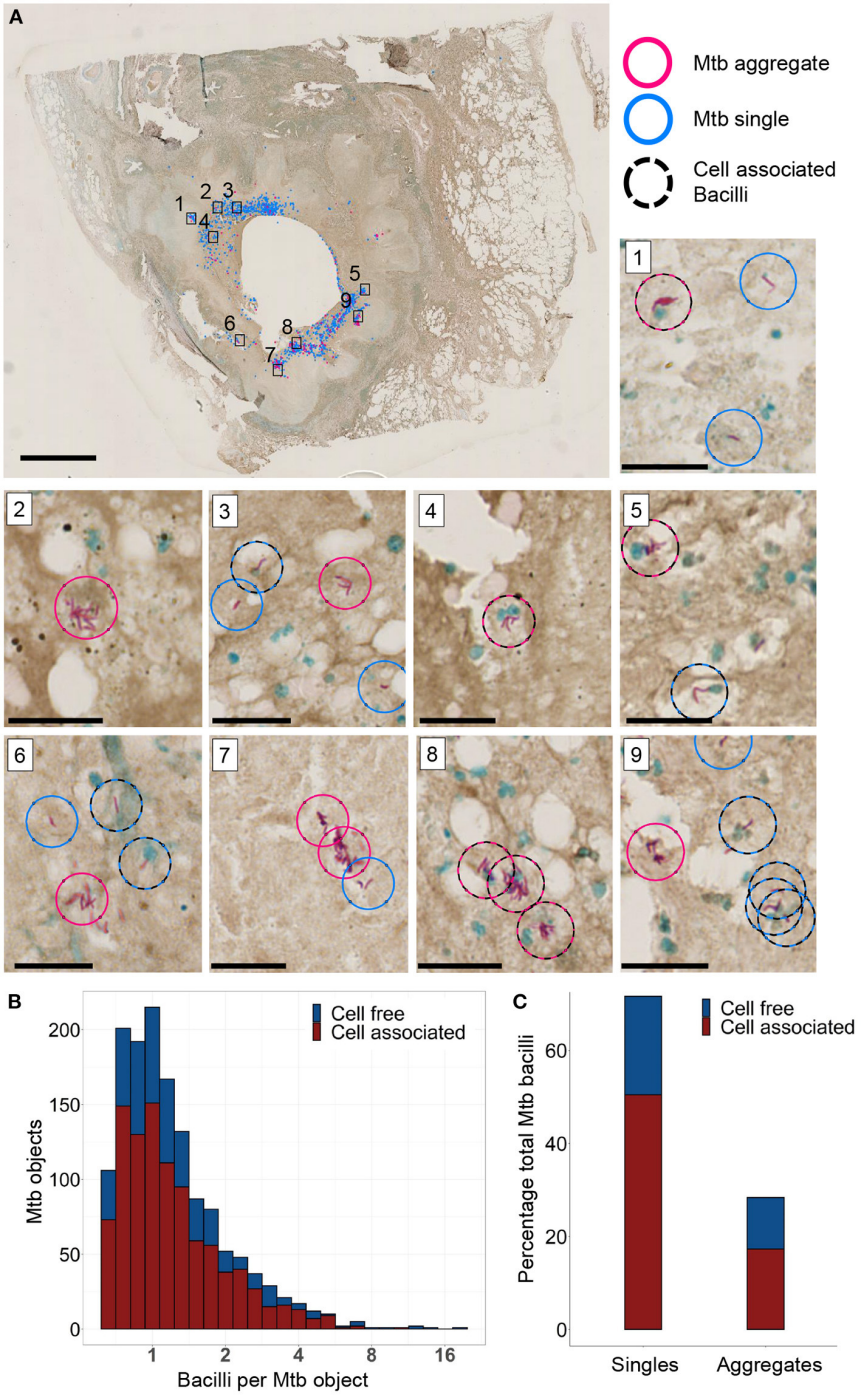
*Mycobacterium tuberculosis* (Mtb) and Mtb aggregates are found near the periphery of a TB cavitary lesion. **(A)** Ziehl-Neelsen stain of a lung section. Aggregated bacilli are highlighted with a red circle and single bacilli with a blue circle. Scale bar is 3 mm. A black dashed circle is overlaid if the Mtb are in close proximity to a cell nucleus (blue stain). Sub-areas 1–9 are magnified in separate panels. Scale bars are 20 μm in the sub-areas. **(B)** Stacked histogram of the number of Mtb objects with varying numbers of Mtb bacilli found to be cell free or cell associated. **(C)** Stacked histogram of the total number of Mtb observed to be single bacilli or aggregates, and were found to be in close association with host cell nuclei.

We analyzed stained sections of lung tissue from a TB-infected individual requiring clinically indicated lung lobe resection to determine if Mtb aggregates were present. Image analysis was performed using a custom image analysis code in Matlab 2019a (see Materials and Methods section) to automatically identify Mtb within the tissue section. Mtb was classified as single or aggregated based on size and tested for cell association by proximity to adjacent cell nuclei (Supplementary Figure 3). Mtb were located around the TB cavity but not elsewhere in the section (Figure 6A). A total of 1420 Mtb objects, containing different numbers of individual bacilli, were detected (Figure 6B). The total number of Mtb bacilli present in all objects was estimated at 2086, based on the average size of an individual bacterium (see Materials and methods section). Note that 151 objects were classified as Mtb aggregates and corresponded to a minimum of 2.4 Mtb bacilli. These accounted for 28% of all detected Mtb bacilli (Figure 6C). Also 993 Mtb objects, 68% of all bacilli, detected were within close proximity of host cell nuclei and were classified as cell associated; 61% of the aggregated and 70% of the single Mtb bacilli were cell associated (Figure 6C). These data support the existence of aggregates in a naturally infected human lung.

## Discussion

In a previous study, we observed that infection of MDM with aggregated viable Mtb was associated with increased replication of the bacilli. This was mediated by macrophage death, which led to robust Mtb replication in the dead infected cell (Mahamed et al., 2017). Here, we investigated the early transcriptional response of MDM to infection by single or aggregated Mtb and observed that infection with aggregates upregulates the TNFα gene network to a greater extent than infection with single or non-aggregated multiple Mtb per cell. More broadly, infection with Mtb aggregates led to differential regulation of a large number of genes, which are not regulated by either single or multiple single Mtb infection. For the most strongly regulated genes, as determined by a combination of both DESeq2 differential expression and PCA analysis, aggregate infection was the condition of highest gene expression. For UHFR1, a gene involved in cell cycle progression and negatively regulated by Mtb infection, expression is lowest in aggregates. While the largest significant difference tended to be between the uninfected and infected macrophages, for almost all strongly regulated genes there was a trend for aggregate infection having the strongest effect, and in TNF and IER3, there was a significant difference relative to infection by multiple single Mtb. There was no significant difference in IER3 regulation between single and multiple single infection. An exception to this trend was regulation of SERPINB2 and TNFAIP6, which showed similar upregulation regardless of infection mode.

We have previously observed that macrophage death was strongly enhanced by aggregate versus single Mtb infection and that cell death due to aggregates happens by non-apoptotic processes (Mahamed et al., 2017). Building on these previous results, in this work we examined whether aggregates need to be composed of live Mtb to elicit this effect. When we infected cells with live versus heat killed aggregates, we observed that aggregates composed of dead Mtb do not significantly elicit cell death, consistent with an active induction of cell death by a mechanism such as a cell necrosis associated toxin (Sun et al., 2015; Tak et al., 2019). Supporting non-apoptotic cell death by aggregate infection, the genes we observed to be upregulated in this study with aggregate infection include IER3, which has been shown to be involved in the negative regulation of apoptotic cell death (Wu et al., 1998; Ribeil et al., 2007).

Possibly related to poorer control of growth of Mtb aggregates and macrophage death, we observed that as per Mtb bacillus, acidification was reduced as aggregate size became larger. We speculate that while overall increased TNF-alpha activation may be deleterious to the survival of the host cell, it has less effect on the aggregated bacillus.

One possibility of how Mtb aggregates may play a role in progression to disease and in TB pathogenesis may be the recruitment of additional phagocytes to the infection site. CCL4 (Bless et al., 2000; Kobayashi, 2008) and CXCL3 (de Oliveira et al., 2016), were upregulated in MDM infected with aggregates. These molecules are chemokines which recruit neutrophils. Neutrophil accumulation in Mtb infection leads to increased inflammation and potential liquefaction and has been shown to be detrimental to the viability of host cells (Nandi and Behar, 2011; Hilda et al., 2020). Aggregated Mtb infection led to neutrophil accumulation in a mouse model (Arias et al., 2020). Death of Mtb-infected neutrophils may also enhance Mtb growth, potentially accelerating bacillary replication in the cells (Mahamed et al., 2017).

We observed that Mtb aggregates occurred at a substantial frequency in the necrotic zone adjacent to the cavity in the lesion. This is one illustration of Mtb aggregates in the lung, but the limitations are that we cannot infer from the section that aggregates are more cytotoxic since the whole region is necrotic and we cannot determine whether aggregates are proliferating. Furthermore, there are more single than aggregated Mtb, by a ratio of about 2:1 and their spatial distributions overlap. Animal models may give more insight into Mtb aggregate effects *in vivo*. A recent study showed increased pathogenicity with aggregate relative to single Mtb infections (Kolloli et al., 2021), complementing the *in vivo* observations in the human lung described here.

In summary, in this study we have found that (1) infection with Mtb aggregates differentially regulates many more genes that are either single Mtb or infection with multiple single Mtb; (2) genes which are most strongly regulated by Mtb tend also to be most strongly regulated by aggregate infection relative to the other infection modes; (3) to mediate killing of macrophages, aggregates need to be composed of live Mtb; (4) aggregated Mtb are less acidified on a per bacillus basis in macrophage phagosomes relative to single Mtb; (5) aggregated Mtb can be found around a cavity in a TB lesion, possibly allowing them to be in the correct location for transmission. Aggregates were recently observed in bio-aerosols of TB patients (Dinkele et al., 2021). If aggregate transmission occurs, the increased virulence of Mtb aggregates may potentially increase the probability of the newly exposed individual to develop active TB disease rather than remain latently infected.

## Data Availability Statement

The datasets presented in this study can be found in online repositories. The names of the repository/repositories and accession number(s) can be found below: https://www.ncbi.nlm.nih.gov/geo/, GSE173560.

## Ethics Statement

Blood was obtained from adult healthy volunteers after written informed consent (University of KwaZulu-Natal Institutional Review Board approval BE022/13). Lung sections were obtained from clinically indicated resection due to TB complications (University of KwaZulu-Natal Institutional Review Board approval BE019/13). Written informed consent was obtained.

## Author Contributions

HR contributed to conception, acquisition, analysis and interpretation of data, as well as drafting and revision. ASi contributed to conception of the study and analysis, interpretation and revision of data. ASi and HR are accountable for the work. CZ, IF, S-HH, KN, YG, AKS, and AJCS contributed to data acquisition. MB, GL, GK, and MN were involved in providing approval for publication content. MB additionally performed some analysis on the data. All authors contributed to the article and approved the submitted version.

## Funding

This study was supported by a Bill and Melinda Gates Foundation Award OPP1116944. IF was supported through a Sub-Saharan African Network for TB/HIV Research Excellence [SANTHE, a DELTAS Africa Initiative (grant DEL-15-006)] fellowship. AKS was supported by the Searle Scholars Program, the Beckman Young Investigator Program, and a Sloan Fellowship in Chemistry.

## Conflict of Interest

The authors declare that the research was conducted in the absence of any commercial or financial relationships that could be construed as a potential conflict of interest.

## Publisher's Note

All claims expressed in this article are solely those of the authors and do not necessarily represent those of their affiliated organizations, or those of the publisher, the editors and the reviewers. Any product that may be evaluated in this article, or claim that may be made by its manufacturer, is not guaranteed or endorsed by the publisher.

## Supplementary Material

Supplementary Figure 1 Quantification of the number of Mtb bacilli per macrophage in flow cytometry data. **(A)** A distribution of bacterial fluorescence, within infected macrophages, was obtained using confocal fluorescence microscopy prior to flow cytometric sorting of macrophages. Single Mtb bacteria were identified in the image and their position within the fluorescence distribution was determined. **(B)** This position was then applied to the matching distribution from cell sorting data to quantify bacteria. Scale bar = 20μm.

Supplementary Figure 2 Transcriptional upregulation and secretion of TNFα and downstream genes with aggregated versus single Mtb infection. **(A)** Normalized transcripts or **(B)** cytokine secretion 3 h post-Mtb infection. MDM were either uninfected (UI), infected with single Mtb culture (SCI) or with aggregated Mtb culture (ACI). Shown are median and IQR of the transcriptional or cytokine response from 15 independent infections of MDM from 5 blood donors. p-values are ^*^ < 0.01; ^**^ < 0.001; ^***^ < 0.0001; ^****^ < 0.00001 as determined by Kruskal-Wallis test with Bonferroni multiple comparison correction.

Supplementary Figure 3 Schematic overview of semi-automated histological image analysis. **(A)** The section was scanned using a Hamamatsu Nanozoomer 2.0 rs slide scanner and exported to **(B)** ImageJ to split large files into smaller image tiles in preparation for processing in Matlab. **(C)** Smaller image tiles were individually loaded into Matlab and **(D)** thresholded in the Mtb channel to create binary masks corresponding to the locations of Mtb bacilli. Each of these individual binary object masks **(E)** were applied to the original RGB image **(F)** to isolate full RGB profiles of the objects. Each of these object profiles was then compared to a reference RGB pattern that matched the RGB profile of stained Mtb **(G)**. Objects that were within the RGB thresholds were added to the database. Objects that succeeded the RGB profile thresholds were then individually manually curated **(H)** and added to the curated database **(I)**.

Supplementary Movie 1 Infection of macrophage with live Mtb aggregates. Monocyte derived macrophages were infected with live Mtb aggregates (red) and incubated with the cell membrane permeability dye DRAQ7 (green). Penetration of DRAQ7 indicates loss of membrane integrity and cell death. Scale bar = 20μm, time is hh:mm.

Supplementary Movie 2 Infection of macrophage with heat-killed Mtb aggregates. Monocyte derived macrophages were infected with heat-killed Mtb aggregates (red) and incubated with the cell membrane permeability dye DRAQ7 (green). Absence of DRAQ7 signal indicates retention of cell wall integrity indicative of a viable cell. Scale bar = 20μm, time is hh:mm.
