# Supplementary material for: Aggregated Mycobacterium tuberculosis Enhances the Inflammatory Response
Source: Front Microbiol. 2021 Dec 2;12:757134. doi: 10.3389/fmicb.2021.757134 (PMC8674758; doi:10.3389/fmicb.2021.757134)
Supplement: Supplementary Figure 1 — Quantification of the number of Mtb bacilli per macrophage in flow cytometry data. (A) A distribution of bacterial fluorescence, within infected macrophages, was obtained using confocal fluorescence microscopy prior to flow cytometric sorting of macrophages. Single Mtb bacteria were identified in the image and their position within the fluorescence distribution was determined. (B) This position was then applied to the matching distribution from cell sorting data to quantify bacteria. Scale bar = 20μm. [file Image_1.pdf]

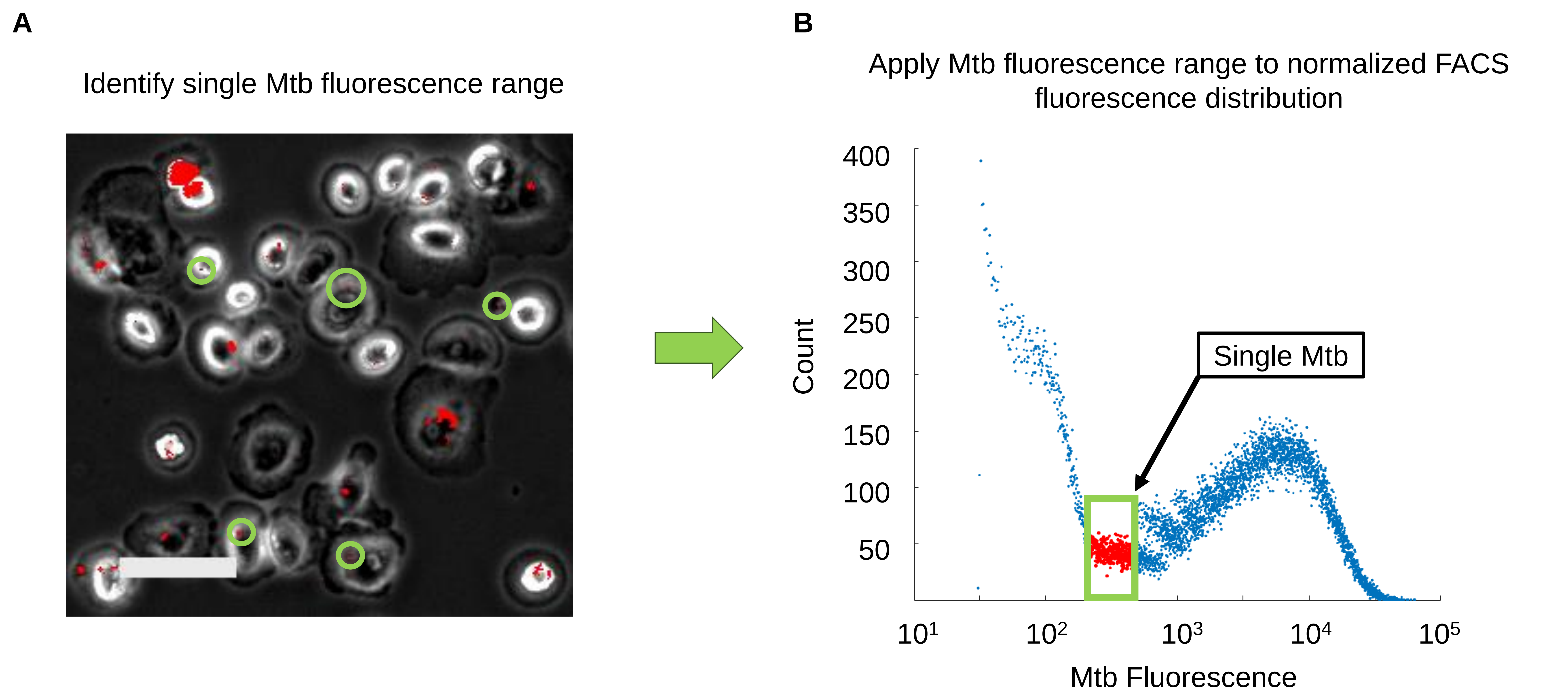

A
Identify single Mtb fluorescence range
B
Apply Mtb fluorescence range to normalized FACS fluorescence distribution
400
350
300
250
200
150
100
50
Count
Single Mtb
$10^1$
$10^2$
$10^3$
$10^4$
$10^5$
Mtb Fluorescence
