# Supplementary figures and images for: Aggregated Mycobacterium tuberculosis Enhances the Inflammatory Response
Source: Front Microbiol. 2021 Dec 2;12:757134. doi: 10.3389/fmicb.2021.757134 (PMC8674758; doi:10.3389/fmicb.2021.757134)

A

## Gene expression

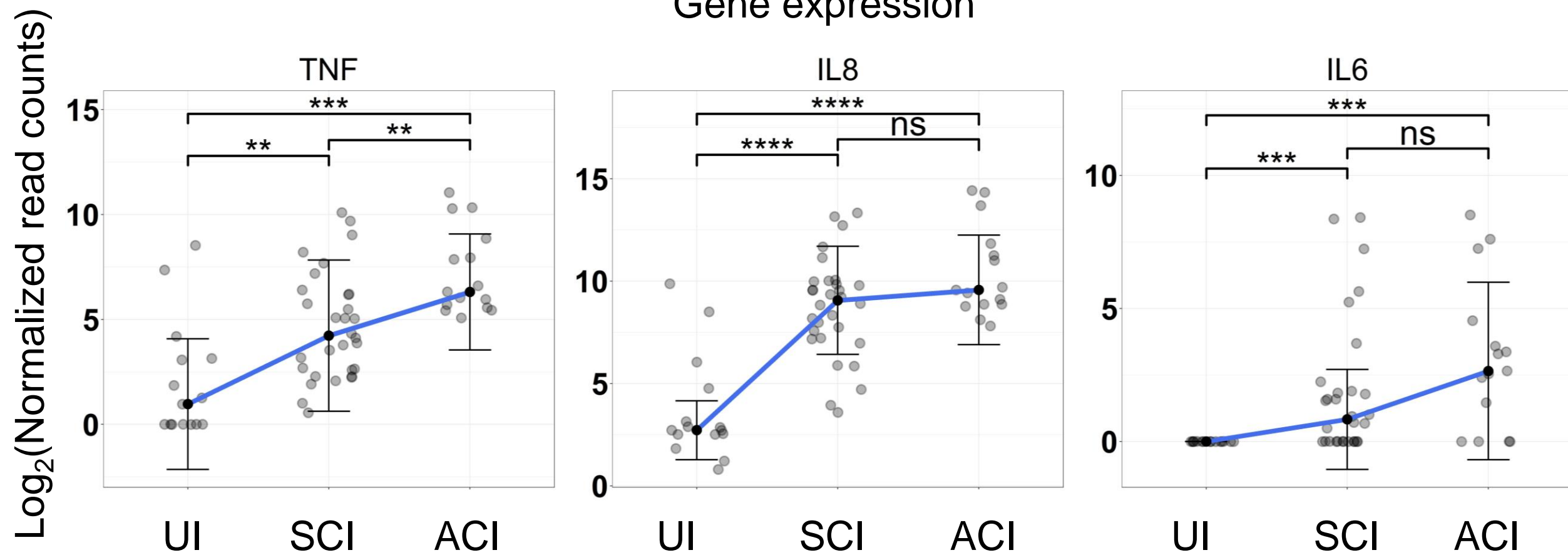

B

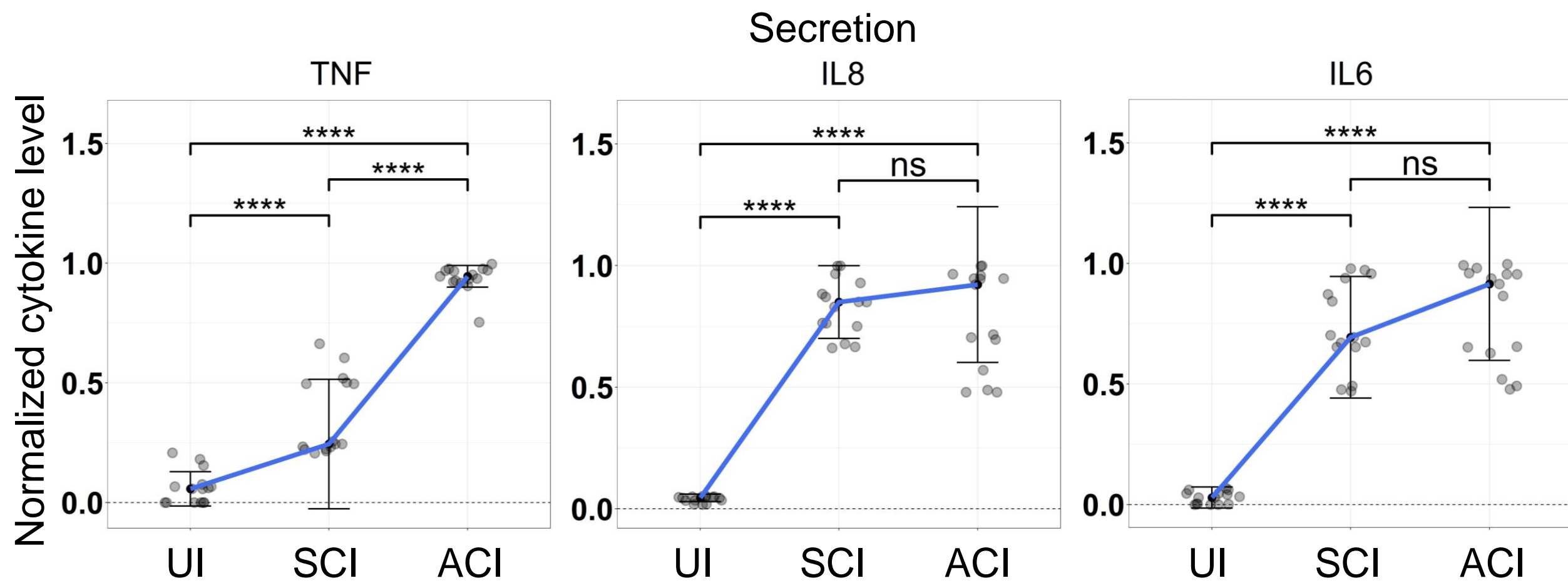

Supplement: Supplementary Figure 2 — Transcriptional upregulation and secretion of TNFα and downstream genes with aggregated versus single Mtb infection. (A) Normalized transcripts or (B) cytokine secretion 3 h post-Mtb infection. MDM were either uninfected (UI), infected with single Mtb culture (SCI) or with aggregated Mtb culture (ACI). Shown are median and IQR of the transcriptional or cytokine response from 15 independent infections of MDM from 5 blood donors. p-values are * < 0.01; ** < 0.001; *** < 0.0001; **** < 0.00001 as determined by Kruskal-Wallis test with Bonferroni multiple comparison correction. [file Image_2.pdf]

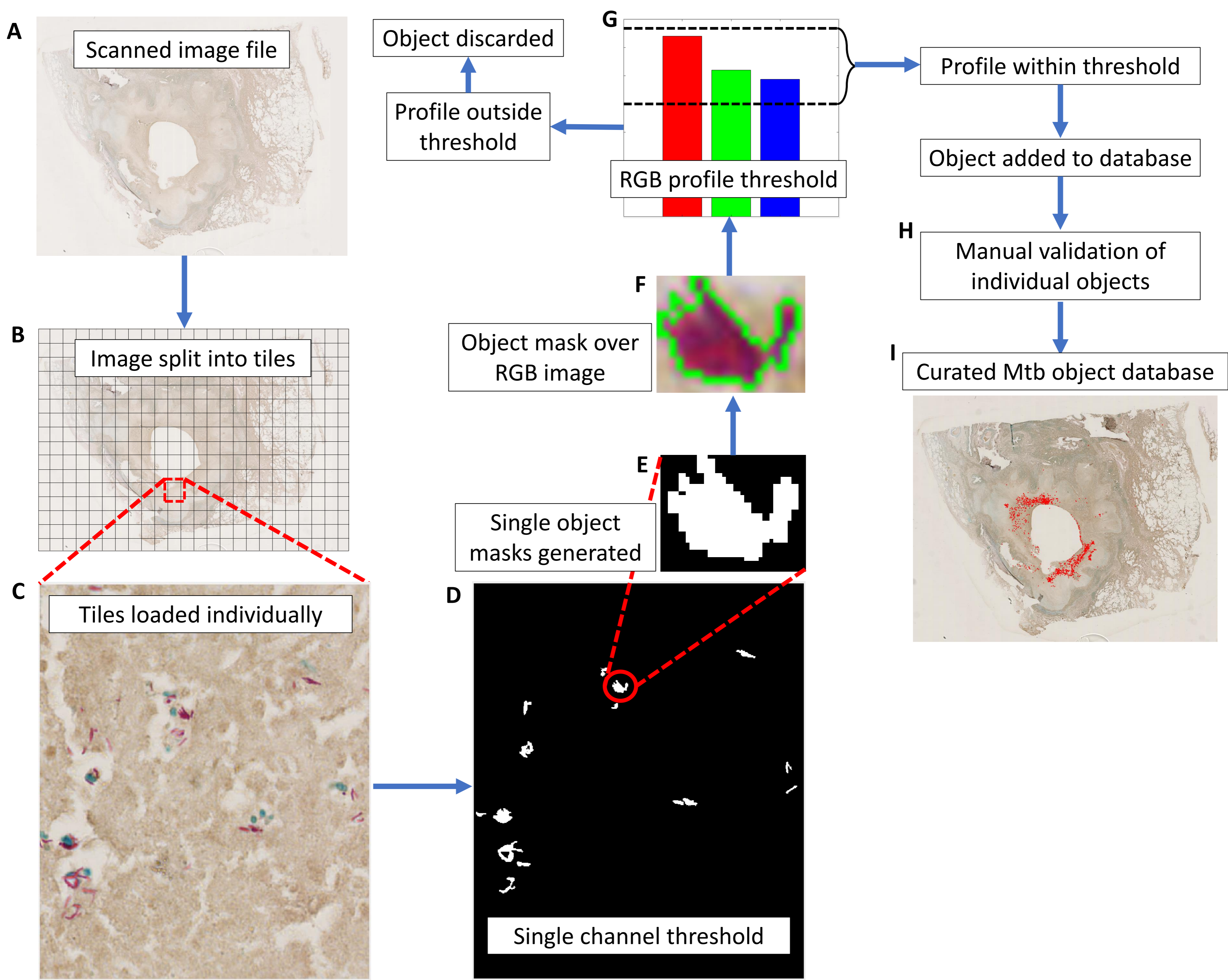

Supplement: Supplementary Figure 3 — Schematic overview of semi-automated histological image analysis. (A) The section was scanned using a Hamamatsu Nanozoomer 2.0 rs slide scanner and exported to (B) ImageJ to split large files into smaller image tiles in preparation for processing in Matlab. (C) Smaller image tiles were individually loaded into Matlab and (D) thresholded in the Mtb channel to create binary masks corresponding to the locations of Mtb bacilli. Each of these individual binary object masks (E) were applied to the original RGB image (F) to isolate full RGB profiles of the objects. Each of these object profiles was then compared to a reference RGB pattern that matched the RGB profile of stained Mtb (G). Objects that were within the RGB thresholds were added to the database. Objects that succeeded the RGB profile thresholds were then individually manually curated (H) and added to the curated database (I). [file Image_3.pdf]
