# Supplementary material for: Aggregated Mycobacterium tuberculosis Enhances the Inflammatory Response
Source: Front Microbiol. 2021 Dec 2;12:757134. doi: 10.3389/fmicb.2021.757134 (PMC8674758; doi:10.3389/fmicb.2021.757134)
Supplement: Supplementary file 6 [file Table_1.docx]

Table 1: GSEA differentially regulated gene sets between infection conditions at Nominal p-value and FDR < 0.05

| Gene set | Gene set size | NES | Nominal P-value | FDR q-value |
| --- | --- | --- | --- | --- |
| Aggregate vs. Uninfected | | | | |
| tnfα signaling via nfκb | 200 | 2,81 | < 0,001 | < 0,001 |
| inflammatory response | 200 | 2,46 | < 0,001 | < 0,001 |
| allograft rejection | 200 | 1,63 | < 0,001 | 0,010 |
| interferon gamma response | 200 | 1,61 | < 0,001 | 0,009 |
| il6 jak stat3 signaling | 87 | 1,61 | < 0,001 | 0,007 |
| hypoxia | 200 | 1,59 | < 0,001 | 0,008 |
| cholesterol homeostasis | 74 | 1,46 | 0,009 | 0,038 |
| apoptosis | 161 | 1,44 | < 0,001 | 0,038 |
| kras signaling up | 200 | 1,43 | 0,004 | 0,037 |
| complement | 200 | 1,39 | 0,003 | 0,046 |
| Multiple vs. Uninfected | | | | |
| tnfα signaling via nfκb | 200 | 2,59 | < 0,001 | < 0,001 |
| inflammatory response | 200 | 2,24 | < 0,001 | < 0,001 |
| il6 jak stat3 signaling | 87 | 1,81 | < 0,001 | < 0,001 |
| complement | 200 | 1,59 | < 0,001 | 0,014 |
| interferon gamma response | 200 | 1,58 | < 0,001 | 0,013 |
| allograft rejection | 200 | 1,57 | < 0,001 | 0,012 |
| apoptosis | 161 | 1,45 | 0,004 | 0,043 |
| Single vs Uninfected | | | | |
| tnfα signaling via nfκb | 200 | 2,42 | < 0,001 | < 0,001 |
| inflammatory response | 200 | 1,83 | < 0,001 | < 0,001 |
| cholesterol homeostasis | 74 | 1,57 | 0,011 | 0,032 |
| notch signaling | 32 | 1,51 | 0,025 | 0,038 |
| complement | 200 | 1,48 | < 0,001 | 0,040 |
| Multiple vs. Single | | | | |
| inflammatory response | 200 | 1,73 | < 0,001 | 0,004 |
| tnfα signaling via nfκb | 200 | 1,68 | < 0,001 | 0,004 |
| il6 jak stat3 signaling | 87 | 1,63 | < 0,001 | 0,005 |
| e2f targets | 200 | 1,46 | 0,001 | 0,040 |
| Aggregate vs. Single | | | | |
| tnfα signaling via nfκb | 200 | 2,24 | < 0,001 | < 0,001 |
| inflammatory response | 200 | 1,87 | < 0,001 | 0,001 |
| Aggregate vs. Multiple | | | | |
| tnfα signaling via nfκb | 200 | 2,07 | < 0,001 | < 0,001 |
