# Supplementary material for: Aggregated Mycobacterium tuberculosis Enhances the Inflammatory Response
Source: Front Microbiol. 2021 Dec 2;12:757134. doi: 10.3389/fmicb.2021.757134 (PMC8674758; doi:10.3389/fmicb.2021.757134)
Supplement: Supplementary file 7 [file Table_2.docx]

Table 2: DESeq2 differentially regulated genes between infection conditions at adjusted p-value < 0.01

| Gene | Mean reads | log_2_ Fold Change | Adjusted p |
| --- | --- | --- | --- |
| Aggregate relative to Uninfected | | | |
| CCL4L1 | 117 | 6,29 | <0,0001 |
| CCL4L2 | 117 | 6,29 | <0,0001 |
| IL1B | 923 | 5,06 | <0,0001 |
| IL8 | 1886 | 6,16 | <0,0001 |
| CCL4 | 374 | 5,96 | <0,0001 |
| SOD2 | 847 | 2,58 | <0,0001 |
| SERPINB2 | 53 | 6,39 | <0,0001 |
| TNFAIP6 | 232 | 5,40 | <0,0001 |
| CCL3 | 1098 | 2,82 | <0,0001 |
| ICAM1 | 702 | 2,04 | <0,0001 |
| HSPA1A | 64 | 2,32 | <0,0001 |
| EHD1 | 206 | 2,03 | <0,0001 |
| CCL20 | 43 | 6,62 | <0,0001 |
| ZC3H12A | 73 | 2,58 | <0,0001 |
| CXCL3 | 175 | 5,30 | <0,0001 |
| MFSD2A | 140 | 1,67 | <0,0001 |
| PIM1 | 346 | 1,28 | <0,0001 |
| SLC2A6 | 170 | 1,93 | <0,0001 |
| INHBA | 42 | 3,14 | <0,0001 |
| BTG2 | 316 | 1,10 | <0,0001 |
| OSGIN1 | 85 | 1,41 | <0,0001 |
| AMPD3 | 126 | 1,60 | <0,0001 |
| TNF | 172 | 4,60 | <0,0001 |
| C15orf48 | 557 | 1,28 | <0,0001 |
| CXCL2 | 219 | 4,97 | <0,0001 |
| NFE2L2 | 300 | 0,81 | <0,0001 |
| CCL3L1 | 276 | 8,59 | <0,0001 |
| CXCL1 | 85 | 5,35 | <0,0001 |
| CD274 | 125 | 1,51 | <0,0001 |
| IER3 | 65 | 2,77 | <0,0001 |
| PTGS2 | 21 | 3,78 | <0,0001 |
| NCK2 | 16 | 2,40 | <0,0001 |
| NAMPT | 154 | 1,64 | <0,0001 |
| TRAF1 | 80 | 1,69 | <0,0001 |
| LIF | 31 | 2,06 | <0,0001 |
| HLA-DQA1 | 345 | 1,48 | <0,0001 |
| SQSTM1 | 2922 | 0,88 | <0,0001 |
| IRAK2 | 41 | 2,19 | <0,0001 |
| BID | 205 | 0,98 | <0,0001 |
| IL7R | 70 | 2,79 | <0,0001 |
| ADORA2A | 14 | 4,73 | <0,0001 |
| IL6 | 29 | 5,63 | <0,0001 |
| KYNU | 455 | 1,17 | <0,0001 |
| PDE4DIP | 315 | 0,72 | <0,0001 |
| KDM6B | 9 | 2,89 | <0,0001 |
| MAP3K8 | 26 | 1,50 | <0,0001 |
| RNF144B | 37 | 1,70 | <0,0001 |
| DRAM1 | 166 | 1,04 | <0,0001 |
| CSF1 | 437 | 1,13 | <0,001 |
| MSANTD3 | 30 | 1,49 | <0,001 |
| MSC | 305 | 0,93 | <0,001 |
| AMZ1 | 68 | 1,61 | <0,001 |
| G0S2 | 30 | 2,75 | <0,001 |
| GPR35 | 18 | 2,60 | <0,001 |
| TNFAIP3 | 151 | 1,84 | <0,001 |
| TRAF3 | 69 | 1,17 | <0,001 |
| MTHFD2 | 446 | 0,65 | <0,001 |
| HCK | 309 | 0,64 | <0,001 |
| RILPL2 | 94 | 0,91 | <0,001 |
| HSPA1B | 19 | 2,26 | <0,001 |
| SLC2A3 | 231 | 1,14 | <0,001 |
| ATP2B1 | 55 | 1,24 | <0,001 |
| GEM | 70 | 1,08 | <0,001 |
| HMOX1 | 573 | 0,63 | <0,01 |
| B4GALT1 | 391 | 1,05 | <0,01 |
| PPP1R15A | 444 | 0,96 | <0,01 |
| IFIT2 | 27 | 1,72 | <0,01 |
| STX11 | 114 | 0,79 | <0,01 |
| MAP2K3 | 525 | 0,71 | <0,01 |
| CD80 | 4 | 3,03 | <0,01 |
| CES1 | 237 | 1,54 | <0,01 |
| RIN2 | 30 | -1,39 | <0,01 |
| TREM1 | 37 | 1,94 | <0,01 |
| IL1A | 26 | 2,66 | <0,01 |
| CCRL2 | 122 | 0,87 | <0,01 |
| CD180 | 41 | -1,27 | <0,01 |
| PDK4 | 126 | -1,15 | <0,01 |
| CYP27B1 | 63 | 1,01 | <0,01 |
| TNFSF15 | 45 | 1,96 | <0,01 |
| EDN1 | 6 | 3,27 | <0,01 |
| SDC4 | 239 | 0,71 | <0,01 |
| HIVEP2 | 21 | 1,85 | <0,01 |
| NFKBIA | 397 | 1,26 | <0,01 |
| CD83 | 972 | 0,72 | <0,01 |
| LINC00674 | 35 | -0,99 | <0,01 |
| DENND5A | 43 | 1,23 | <0,01 |
| USF1 | 19 | 1,15 | <0,01 |
| MORC3 | 62 | 0,96 | <0,01 |
| Multiple relative to Uninfected | | | |
| IL1B | 923 | 4,33 | <0,0001 |
| SERPINB2 | 53 | 6,27 | <0,0001 |
| SOD2 | 847 | 2,40 | <0,0001 |
| CCL4L1 | 117 | 4,81 | <0,0001 |
| CCL4L2 | 117 | 4,81 | <0,0001 |
| IL8 | 1886 | 5,06 | <0,0001 |
| TNFAIP6 | 232 | 5,01 | <0,0001 |
| SLC2A6 | 170 | 2,16 | <0,0001 |
| CCL4 | 374 | 4,26 | <0,0001 |
| C15orf48 | 557 | 1,49 | <0,0001 |
| PIM1 | 346 | 1,28 | <0,0001 |
| BTG2 | 316 | 1,11 | <0,0001 |
| EHD1 | 206 | 1,73 | <0,0001 |
| AMPD3 | 126 | 1,54 | <0,0001 |
| HCK | 309 | 0,87 | <0,0001 |
| BID | 205 | 1,08 | <0,0001 |
| IL7R | 70 | 3,08 | <0,0001 |
| CCL3 | 1098 | 1,87 | <0,0001 |
| ICAM1 | 702 | 1,44 | <0,0001 |
| OSGIN1 | 85 | 1,22 | <0,0001 |
| SQSTM1 | 2922 | 0,85 | <0,0001 |
| CXCL1 | 85 | 4,42 | <0,0001 |
| TNF | 172 | 3,73 | <0,0001 |
| CD274 | 125 | 1,30 | <0,0001 |
| CXCL3 | 175 | 3,54 | <0,0001 |
| KYNU | 455 | 1,13 | <0,0001 |
| GPR35 | 18 | 2,68 | <0,001 |
| CCL20 | 43 | 4,16 | <0,001 |
| NCK2 | 16 | 1,90 | <0,001 |
| PDE4DIP | 315 | 0,63 | <0,001 |
| NAMPT | 154 | 1,25 | <0,001 |
| ACSL1 | 2158 | 1,30 | <0,01 |
| TRAF1 | 80 | 1,29 | <0,01 |
| NCF1 | 30 | 1,74 | <0,01 |
| MSC | 305 | 0,83 | <0,01 |
| CCL3L1 | 276 | 5,57 | <0,01 |
| INHBA | 42 | 1,90 | <0,01 |
| HLA-DQA1 | 345 | 1,09 | <0,01 |
| DENND5A | 43 | 1,31 | <0,01 |
| NCAPH | 192 | -0,81 | <0,01 |
| SLC7A11 | 398 | 1,11 | <0,01 |
| NFE2L2 | 300 | 0,50 | <0,01 |
| DRAM1 | 166 | 0,85 | <0,01 |
| IFNGR2 | 275 | 0,93 | <0,01 |
| RILPL2 | 94 | 0,78 | <0,01 |
| CES1 | 237 | 1,43 | <0,01 |
| MTHFD2 | 446 | 0,55 | <0,01 |
| GPR68 | 53 | 1,09 | <0,01 |
| AMZ1 | 68 | 1,35 | <0,01 |
| MFSD2A | 140 | 0,91 | <0,01 |
| IVNS1ABP | 435 | -0,52 | <0,01 |
| Single relative to Uninfected | | | |
| SERPINB2 | 53 | 5,63 | <0,0001 |
| TNFAIP6 | 232 | 4,83 | <0,0001 |
| SOD2 | 847 | 1,94 | <0,0001 |
| IL1B | 923 | 3,27 | <0,0001 |
| CCL4L1 | 117 | 3,75 | <0,0001 |
| CCL4L2 | 117 | 3,75 | <0,0001 |
| IL8 | 1886 | 3,85 | <0,0001 |
| SLC2A6 | 170 | 1,71 | <0,0001 |
| PIM1 | 346 | 1,09 | <0,0001 |
| AMPD3 | 126 | 1,39 | <0,0001 |
| EHD1 | 206 | 1,47 | <0,0001 |
| C15orf48 | 557 | 1,05 | <0,0001 |
| CCL4 | 374 | 2,94 | <0,0001 |
| KYNU | 455 | 1,23 | <0,0001 |
| BTG2 | 316 | 0,82 | <0,0001 |
| ICAM1 | 702 | 1,24 | <0,0001 |
| BID | 205 | 0,87 | <0,001 |
| HCK | 309 | 0,67 | <0,001 |
| SQSTM1 | 2922 | 0,74 | <0,001 |
| OSGIN1 | 85 | 1,01 | <0,001 |
| TNIP1 | 38 | 1,40 | <0,001 |
| MSC | 305 | 0,86 | <0,01 |
| CCL3 | 1098 | 1,28 | <0,01 |
| PDE4DIP | 315 | 0,57 | <0,01 |
| TRAF1 | 80 | 1,20 | <0,01 |
| HMOX1 | 573 | 0,58 | <0,01 |
| Aggregate relative to Single | | | |
| CXCL2 | 219 | 4,77 | <0,0001 |
| CCL4 | 374 | 3,03 | <0,0001 |
| CCL4L1 | 117 | 2,54 | <0,0001 |
| CCL4L2 | 117 | 2,54 | <0,0001 |
| TNF | 172 | 3,97 | <0,0001 |
| HSPA1A | 64 | 1,67 | <0,0001 |
| CXCL3 | 175 | 3,63 | <0,0001 |
| ZC3H12A | 73 | 1,68 | <0,001 |
| CCL3 | 1098 | 1,54 | <0,001 |
| IER3 | 65 | 2,20 | <0,001 |
| MAP3K8 | 26 | 1,43 | <0,001 |
| CCL2 | 103 | 1,40 | <0,01 |
| CXCL1 | 85 | 3,55 | <0,01 |
| IRAK2 | 41 | 1,76 | <0,01 |
| INHBA | 42 | 1,86 | <0,01 |
| IL8 | 1886 | 2,31 | <0,01 |
| Aggregate relative to Multiple | | | |
| HSPA1A | 64 | 1,51 | <0,001 |
